# Patient-prosthesis mismatch in patients with aortic valve replacement

**DOI:** 10.1007/s11748-013-0216-6

**Published:** 2013-02-13

**Authors:** Yuichiro Kaminishi, Yoshio Misawa, Junjiro Kobayashi, Hiroaki Konishi, Hiroaki Miyata, Noboru Motomura, Shin-ichi Takamoto, Japan Cardiovascular Surgery Database Organization

**Affiliations:** 1 Division of Cardiovascular Surgery, Jichi Medical University, 3311-1 Yakushiji, Shimotsuke, Tochigi 329-0498 Japan; 2 Japan Cardiovascular Surgery Database Organization, 2-26-9 Hongo, Bunnkyo-ku, Tokyo 113-0033 Japan

**Keywords:** Aortic valve replacement, Prosthesis, Mortality, Hemodynamics

## Abstract

**Objective:**

Patient-prosthesis mismatch (PPM) may affect clinical outcomes in patients with aortic valve replacement (AVR). We retrospectively examined the PPM in patients with isolated AVR in the Japan Adult Cardiovascular Surgery Database (JACVSD).

**Methods:**

We examined all patients with isolated AVR between January 1, 2008 and December 31, 2009. The JACVSD data collection form has a total of 255 variables. We defined PPM as an effective orifice area index of ≤0.85 m^2^/cm^2^.

**Results:**

PPM was observed in 306 of 3,609 cases analyzed, PPM rate was 8.5 %. Body surface area was larger and body mass index was higher in the PPM group than the non-PPM group (*P* < 0.001). Patients with PPM were older (*P* = 0.001) and had a higher prevalence of diabetes (*P* = 0.004), dyslipidemia (*P* < 0.001), hypertension (*P* < 0.001), cerebrovascular disease (*P* = 0.031), old myocardial infarction (*P* = 0.006), previous percutaneous coronary artery intervention (*P* = 0.001), coronary artery disease (*P* = 0.018), and aortic valve stenosis (*P* < 0.001). Perioperative blood transfusion (*P* < 0.001) and dialysis (*P* = 0.005) were more frequent in the PPM group. Postoperative ventilation (*P* = 0.004) and intensive care unit stay (*P* = 0.004) were significantly longer in the PPM group.

**Conclusions:**

Age, aortic valve stenosis, dyslipidemia, hypertension, old myocardial infarction, previous percutaneous coronary artery intervention, diabetes mellitus, cerebrovascular disease, and high body mass index were the risk factors for PPM. PPM was not an independent risk factor for short-term mortality.

## Introduction

Patient-prosthesis mismatch (PPM) may affect clinical outcomes in patients with aortic valve replacement (AVR). Some clinical studies show differences in early and late outcomes in patients with PPM, but many are single-center studies. Prosthetic heart valves have a limited effective orifice area (EOA), although supra-annular type prostheses with a larger EOA are commercially available. People in the Eastern countries are physically smaller than those in the Western countries, and it is not always easy to implant a prosthetic valve of an adequate size in a smaller patient. Thus, the influence of PPM on clinical results after AVR should be clarified. We retrospectively examined PPM in patients with isolated AVR in Japan.

## Methods

### Study population

The Japan Adult Cardiovascular Surgery Database (JACVSD) was initiated in 2000 to evaluate surgical outcomes after cardiovascular procedures in many centers throughout Japan. The JACVSD currently captures clinical information from nearly half of all the Japanese hospitals performing cardiovascular surgery. The data collection form has a total of 255 variables (definitions are available online at http://www.jacvsd.umin.jp), and these are almost identical to those of the Society of Thoracic Surgeons (STS) National Database (definitions are available online at http://sts.org). The JACVSD has developed a software for the web-based data collection system that enables the data manager of each participating hospital to submit their data electronically to the central office. Although participation in the JACVSD is voluntary, data completeness is a high priority. Accuracy of submitted data was maintained by data auditing, which involved monthly visits by administrative staff to participating hospitals to check data against clinical records. Validity of data was further confirmed by an independent comparison of the volume of cardiac surgery at specific hospitals entered in the JACVSD, compared with that reported in the Japanese Association for Thoracic Surgery annual survey [1].

We examined all patients with isolated AVR between January 1, 2008 and December 31, 2009. First, the JACVSD records that had been obtained without informed consent from the patient were excluded. Records with missing age (or age out of range), missing sex, or missing 30-day status were also excluded. After this data cleaning, the population for this analysis consisted of 3,609 cases from 167 participating institutes throughout Japan. Short-term mortality was defined as death from any cause within 30 days after operation, if the patient was discharged from hospital, or death at any time if the patient was not discharged. Using the definition from a previous study [2, 3], major morbidity was defined as any of the following five postoperative in-hospital complications: stroke, re-operation for any reason, mechanical ventilation for more than 24 h after surgery, renal failure, or deep sternal wound infection.

### Statistical analysis

The predicted EOA was obtained from the catalog of each model and size of prosthesis implanted (Table 1). The EOA index (EOAI) was determined by the EOA of the implanted prosthetic valve (cm^2^) divided by the patient’s preoperative body surface area (m^2^). PPM was defined as an EOAI of ≤0.85 as described and validated in recent studies [4–6].

**Table 1:** Normal reference values of effective orifice areas for the prosthetic valves

	*n* ^a^	(%)	Prosthetic valve size (mm)					
			17	19	21	23	25	27
Stented bioprosthetic valves								
Carpentier–Edwards PERIMOUNT pericardial Magna bioprosthesis	1214	33.6		1.58	1.90	2.07	2.33	–
Carpentier–Edwards PERIMOUNT pericardial bioprosthesis	722	20		1.24	1.45	1.63	–	–
Carpentier–Edwards standard porcine bioprosthesis	67	1.9		0.85	1.48	1.69	1.94	2.25
Carpentier–Edwards supra-annular aortic porcine bioprosthesis	18	0.5		1.10	1.40	1.50	1.70	1.90
Carpentier–Edwards PERIMOUNT plus pericardial bioprosthesis	2	0.06		1.10	1.40	1.50	1.70	1.90
Medtronic intact porcine bioprosthesis	1	0.03		0.85	1.02	1.27	1.40	1.66
Stentless bioprosthetic valves								
Medtronic freestyle stentless porcine bioprosthesis—subcoronary	7	0.2		1.20	1.42	1.70	2.15	2.49
Mechanical prosthesis								
St. Jude medical regent valve	679	18.8	1.30	1.70	2.00	2.50	2.60	3.50
ATS mechanical prosthesis^b^	313	8.6		1.20	1.50	1.70	2.10	2.50
MCRI On-X mechanical prosthesis	199	5.5		1.50	1.70	2.00	2.40	3.20
St. Jude Medical mechanical heart valve	130	3.6		1.00	1.30	1.60	1.80	2.40
St. Jude Medical Masters Series mechanical heart valve	66	1.8		1.00	1.30	1.60	1.80	2.40
CarboMedics standard aortic valve	51	1.4		1.00	1.30	1.60	1.80	2.40
CarboMedics reduced cuff aortic valve	47	1.3		1.00	1.30	1.60	1.80	2.40
St. Jude Medical Mechanical Heart Valve Hemodynamic Plus Series	23	0.64	1.00	1.30	1.60	1.80	2.40	2.70
Sorin Bicarbon (Baxter Mira) mechanical prosthesis slimline	22	0.61	1.01	1.50	1.90			
CarboMedics Top-Hat supra-annular aortic valve	21	0.58		1.00	1.30	1.60	1.80	2.40
CarboMedics mechanical prosthesis	19	0.53		1.00	1.30	1.60	1.80	2.40
CarboMedics small adult aortic and mitral valves	7	0.19	1.00					
Edwards Tekna mechanical prosthesis	1	0.03		0.90	1.20	1.30	1.40	**–**

^a^Number of patients (%) in the present study

^b^ATS prosthetic valve includes two series of AP and the Standard series. EOA of AP18 is equivalent to Standard 21, AP20 is equivalent to Standard 23

We examined the differences between PPM and non-PPM groups using bivariate tests: the Fisher’s exact test and Chi-squared test for categorical covariates, and the unpaired *t* test or Wilcoxon rank-sum test for continuous covariates. Descriptive data are expressed as mean ± 1 standard deviation. The level of statistical significance was set at 0.05 (two-sided).

To examine effects of PPM, we conducted multivariate logistic regression analysis for each outcome [7]. In this analysis, each outcome was entered as an independent variable (short-term mortality, major morbidity), and preoperative patient risk factors in the JACVSD valve risk models were entered as dependent variables [8].

## Results

Based on preoperative and operative data and the aforementioned definitions, 8.5 % of patients (306/3,609) had PPM. Comparisons between the PPM and the non-PPM groups are presented in Table 2. Body surface area (BSA) was larger and body mass index (BMI) was higher in the PPM group than the non-PPM group (*P* < 0.001). Patients with PPM were older (*P* = 0.001) and had a higher prevalence of diabetes (*P* = 0.004), dyslipidemia (*P* < 0.001), hypertension (*P* < 0.001), cerebrovascular disease (CVD *P* = 0.031), old myocardial infarction (*P* = 0.006), previous percutaneous coronary artery intervention (*P* = 0.001), three-vessel coronary artery disease (*P* = 0.018), and predominant aortic valve stenosis (*P* < 0.001). Patients in the non-PPM group had a higher prevalence of moderate or severe aortic insufficiency (*P* < 0.001).

**Table 2:** Preoperative and postoperative data

Variable	Non-PPM (*n* = 3,303)	PPM (*n* = 306)	*p* value
Preoperative data			
Age	68 ± 12	70 ± 10	0.001*
Female	1505 (45.6)	156 (51.0)	0.072
Body surface area, m2	1.55 ± 0.19	1.61 ± 0.18	<0.001*
Body mass index	22.7 ± 3.56	24.2 ± 3.64	<0.001*
Body mass index > 25	100 (3.0)	20 (6.5)	<0.001*
NHYA functional class ≧III	638 (19.3)	71 (23.2)	0.114
Smoking	1118 (33.8)	107 (35.0)	0.705
Hypertension	2090 (63.3)	224 (73.2)	<0.001*
Dyslipidemia	1192 (33.1)	140 (45.8)	<0.001*
Diabetes	543 (16.4)	71 (23.2)	0.004*
Renal failure	347 (10.5)	22 (7.2)	0.075
Dialysis	226 (6.8)	13 (4.2)	0.092
Cerebrovascular disease	239 (7.2)	33 (10.8)	0.031*
Infective endocarditis	186 (5.6)	19 (6.2)	0.698
Chronic lung disease	91 (2.8)	9 (2.9)	0.855
Peripheral arterial disease	172 (5.2)	13 (4.2)	0.587
Thoracic aortic aneurysm	91 (2.8)	10 (3.3)	0.585
Percutaneous coronary intervention	141 (4.3)	28 (9.2)	0.001*
Old myocardial infarction	55 (1.7)	13 (4.2)	0.006*
Angina pectoris	185 (5.6)	26 (8.5)	0.055
Coronary 1 vessel disease	145 (4.4)	19 (6.2)	0.15
Coronary 2 vessel disease	63 (1.9)	11 (3.6)	0.056
Coronary 3 vessel disease	26 (0.8)	7 (2.3)	0.018*
Predominant aortic valve stenosis	2062 (62.4)	245 (80.1)	<0.001*
Aortic valve insufficiency > 3	13074 (41.6)	78 (25.5)	<0.001*
LV ejection fraction < 30 %	102 (3.1)	11 (3.6)	0.606
Operative data			
Emergent/salvage operation	43 (1.3)	6 (2.0)	0.303
Blood transfusion	2141 (64.8)	233 (76.1)	<0.001*
Re-do sternotomy	146 (4.4)	13 (4.2)	1.00
Outcome			
Re-operation for bleeding	105 (3.2)	12 (3.9)	0.498
Stroke	40 (1.2)	7 (2.3)	0.113
Renal failure	130 (3.9)	20 (6.5)	0.036*
Dialysis	43 (1.3)	11 (3.6)	0.005*
Heart Block	57 (1.7)	1 (0.3)	0.058
Prolonged ventilation	189 (5.7)	31 (10.1)	0.004*
ICU stay > 7 day	157 (4.8)	27 (8.8)	0.004*
Short-term mortality	74 (2.2)	12 (3.9)	0.076

Data are no. of patients (%) or mean ± SD values

*NYHA* New York Heart Association, *LV* indicates left ventricular

* Significant (*P* < 0.05) difference

There were no significant differences between the groups with respect to most of the common operative data, except for perioperative blood transfusion (*P* < 0.001). Postoperative renal failure (*P* = 0.036) and dialysis (*P* = 0.005) were significantly more frequent in the PPM group. Postoperative ventilation (*P* = 0.004) and intensive care unit stay (*P* = 0.004) were significantly longer in the PPM group.

The short-term mortality rate was 3.9 % in the PPM group and 2.2 % in the non-PPM group. There was no significant difference between the groups in short-term mortality (*P* = 0.076).

## Discussion

Many articles referring to PPM have been published. PPM is classified according to EOAI as mild-to-moderate (>0.65 to ≤0.85 cm^2^/m^2^) or severe (<0.65 cm^2^/m^2^). Some studies found that PPM had adverse effects on clinical outcomes, [9–11] and others did not [13–20]. Adverse effects include elevated N-terminal pro B-type natriuretic peptide levels, [9] early stenotic dysfunction of bioprosthetic valves, [10] and delayed left ventricular mass (LVM) regression [11]. Tomoeda et al. [12] found that EOAI should be greater than 0.77 cm^2^/m^2^ for adequate LVM regression, but an increasing number of studies have found that LVM regression was not related to EOAI [13–16]. Some investigators reported that postoperative survival was not significantly different between patients with and without PPM [17–20]. Jamieson et al. [19] analyzed 3,343 cases of AVR and found that the predictors of overall mortality were age, age category, New York Heart Association functional class III/IV, concomitant coronary artery bypass graft surgery, prosthesis type, preoperative congestive heart failure, diabetes mellitus, renal failure, and chronic obstructive pulmonary disease. Furthermore, they conclude that EOAI category was not predictive of overall mortality, early mortality, or late mortality.

Aortic root enlargement is sometimes performed to avoid the PPM, but clinical results including increased surgical risks are controversial [21, 22]. Newly developed prosthetic valves with smaller sewing rings and supra-annular implantation techniques contribute to avoiding the PPM. Not surprisingly, the incidence of mismatch also increases with diminishing prosthesis size, and it is widely recognized that patients with a valve size ≤21 mm tend to have much higher gradients. Nonetheless, it must be emphasized that severe mismatch can also occur in patients receiving a prosthesis size >21 mm and that, ultimately, it is always the relation between prosthesis size and body size, rather than each factor taken separately, that determines the final hemodynamic outcome [5].

PPM was observed in 8.5 % patients who underwent isolated AVR during 2008 and 2009 in Japan. Dumesni and PiBarot [11] reported that the rates of PPM were between 20 and 70 %. The low rate of PPM in this series might be due to the physical differences between Japanese people and other people. PPM may be very rare in patients undergoing AVR; therefore, its clinical significance may be less in Japan than previously hypothesized in the Western countries. Thus, aggressive over-sizing or root enlargement strategies may be unwarranted in Japan.

The fact that mismatch occurs more frequently in patients with stenotic native valves and in older patients is also consistent with this concept because patients with stenotic native valves generally have smaller valvular annuli than those with regurgitant valves, and calcific aortic stenosis is by far the most prevalent lesion in older patients undergoing aortic valve replacement. Elderly patients had a higher rate of PPM. PPM could be justified in some elderly patients with lower metabolic requirements and limited physical activity.

Preoperative risk factors for PPM are related to lifestyle-related diseases. The Japan Society for the Study of Obesity defines obesity as BMI ≥25 kg/m^2^. Our study shows that weight control is mandatory in obese patients to reduce the potential adverse effects of PPM. The prevalence of atherosclerotic aortic valve stenosis has increased over recent years. The risk factors for PPM in this study are consistent with the causes of aortic valve disease. Annular stiffness due to calcification and post-inflammatory changes in patients with aortic valve stenosis might cause an inappropriate valve choice followed by PPM.

The rate of PPM was significantly reduced in Japan during the 2 years studied. New generation prosthetic valves such as the Carpentier-Edwards Magna and the SJM Regent valves might have contributed to this reduction. As the JACVSD records do not include valve implantation techniques (supra-annular or intra-annular position), we cannot determine whether such technical differences contributed to the reduction in the rate of PPM.

There was no difference in early postoperative mortality rates between the two groups, but mechanical ventilation longer than 24 h, renal failure requiring dialysis, and intensive care unit stay longer than 7 days were significantly higher in the PPM group. Postoperative complications including early mortality and major morbidity were significantly more frequent in the PPM group than the non-PPM group, resulting in PPM patients requiring more health resources than non-PPM patients. The high morbidity rates may be caused by postoperative low cardiac output syndrome. As the JACVSD records do not include the treatment details such as catecholamine doses, we cannot determine the causes of the high morbidity rate in the PPM group.

### Study limitations

This is a retrospective study, limited to the evaluation of early clinical outcomes. We have used in vitro manufactures’ EOA, which may overestimate in vivo echocardiographic EOA, and we have no data on postoperative transvalvular gradients. The impact of PPM on the functional outcome following AVR is difficult to evaluate because of the confounding effects of concomitant cardiovascular and non-cardiovascular disease. Perioperative cardiac function may be the most important factor for the outcome. We have neither follow-up data on patient functional status nor follow-up echocardiographic data on EOA or left ventricular mass regression. Further studies should be indicated specifically to examine the effect of mismatch on symptomatic improvement and exercise tolerance after aortic valve replacement.

## Conclusions

This study demonstrates that age, aortic valve stenosis, dyslipidemia, hypertension, old myocardial infarction, history of percutaneous coronary artery intervention, diabetes mellitus, cerebrovascular disease, and high BMI are the risk factors for PPM in patients undergoing isolated AVR. Perioperative blood transfusion, dialysis, and intensive care unit stay longer than 7 days were more frequent in the PPM group. PPM was not an independent risk factor for short-term mortality in patients undergoing AVR in Japan.
